# Fluctuation of Public Interest in COVID-19 in the United States: Retrospective Analysis of Google Trends Search Data

**DOI:** 10.2196/19969

**Published:** 2020-07-17

**Authors:** Iltifat Husain, Blake Briggs, Cedric Lefebvre, David M Cline, Jason P Stopyra, Mary Claire O'Brien, Ramupriya Vaithi, Scott Gilmore, Chase Countryman

**Affiliations:** 1 School of Medicine Wake Forest University Winston-Salem, NC United States; 2 Tuba City Regional Healthcare Tuba City, AZ United States

**Keywords:** Infodemiology, COVID-19, SARS-CoV-2, digital health, Google Trends, trend, internet, public health

## Abstract

**Background:**

In the absence of vaccines and established treatments, nonpharmaceutical interventions (NPIs) are fundamental tools to control coronavirus disease (COVID-19) transmission. NPIs require public interest to be successful. In the United States, there is a lack of published research on the factors that influence public interest in COVID-19. Using Google Trends, we examined the US level of public interest in COVID-19 and how it correlated to testing and with other countries.

**Objective:**

The aim of this study was to determine how public interest in COVID-19 in the United States changed over time and the key factors that drove this change, such as testing. US public interest in COVID-19 was compared to that in countries that have been more successful in their containment and mitigation strategies.

**Methods:**

In this retrospective study, Google Trends was used to analyze the volume of internet searches within the United States relating to COVID-19, focusing on dates between December 31, 2019, and March 24, 2020. The volume of internet searches related to COVID-19 was compared to that in other countries.

**Results:**

Throughout January and February 2020, there was limited search interest in COVID-19 within the United States. Interest declined for the first 21 days of February. A similar decline was seen in geographical regions that were later found to be experiencing undetected community transmission in February. Between March 9 and March 12, 2020, there was a rapid rise in search interest. This rise in search interest was positively correlated with the rise of positive tests for SARS-CoV-2 (6.3, 95% CI −2.9 to 9.7; *P*<.001). Within the United States, it took 52 days for search interest to rise substantially after the first positive case; in countries with more successful outbreak control, search interest rose in less than 15 days.

**Conclusions:**

Containment and mitigation strategies require public interest to be successful. The initial level of COVID-19 public interest in the United States was limited and even decreased during a time when containment and mitigation strategies were being established. A lack of public interest in COVID-19 existed in the United States when containment and mitigation policies were in place. Based on our analysis, it is clear that US policy makers need to develop novel methods of communicating COVID-19 public health initiatives.

## Introduction

Over the past 20 years, two pathologic human coronaviruses (HCoVs) emerged that cause significant morbidity and mortality: severe acute respiratory syndrome coronavirus (SARS-CoV) and Middle East respiratory syndrome coronavirus (MERS-CoV). In December 2019, another pathologic HCoV, severe acute respiratory syndrome coronavirus 2 (SARS-CoV-2), emerged in Wuhan, China, causing coronavirus disease (COVID-19) [1-3]. During the severe acute respiratory syndrome (SARS) epidemic in 2003, 8098 cases and 774 deaths were reported. The cases were concentrated in five countries and regions: China, Taiwan, Hong Kong, Singapore, and Canada. SARS was brought under control in 8 months through syndromic surveillance, prompt isolation of patients, strict quarantine, and community-level quarantine. In contrast, in just 3 months, COVID-19 resulted in more than 2800 deaths and 82000 confirmed cases, and more than 46 countries were affected [4]. COVID-19 is associated with significant morbidity and mortality, with a reported case fatality rate as high as 7.2% [5].

Unfortunately, the epidemiological trajectory of SARS-CoV was a poor predictor of its worldwide impact. While many similarities exist between SARS and COVID-19, clear differences in their transmissibility and severity pyramids alter their epidemiologic trajectories. As many as 81% of patients with confirmed COVID-19 have been reported to have mild disease [6]. This contributes to greater community transmission; thus, the application of traditional public health measures for halting human-to-human transmission is more challenging [5].

In the absence of vaccines and established treatments, nonpharmaceutical interventions (NPIs) such as isolation, quarantine, social distancing, and community containment tools are fundamental tools to control human-to-human transmission [7]. Early and sustained response with NPIs has been shown to reduce transmission of a new contagious pathogen [8]. NPIs achieved improved control of COVID-19 in the Republic of Korea, Hong Kong, and Singapore [9,10].

Implementation of containment strategies for COVID-19 began in the United States in January 2020, with travel restrictions, removal of persons with COVID-19 from the community and into medical facilities, and instructions on mandatory quarantine for people traveling from endemic areas. Once community transmission became evident, the United States shifted from containment to a mitigation strategy in early March [11,12]. Public interest is critical to the effectiveness of containment and mitigation strategies alike; indeed, the first confirmed patient with COVID-19 in the US did not have severe symptoms initially but sought evaluation on January 19 after seeing a health alert from the US Centers for Disease Control and Prevention (CDC) [13].

While studies have been performed on public interest within China and Taiwan in the early days of their respective outbreaks, there is a lack of published research of US public interest in COVID-19 during the early containment and mitigation periods [14,15].

When discussing public interest, communication platforms are at the forefront. Since the 1990s, digital media has become the dominant means of communication worldwide [16]. More than 90% of the US population actively uses the internet in their daily lives. Google is the most popular internet search engine in the world. It is also the most popular search engine within the United States, with a search engine market share of 88.2% [17]. Google Trends, a real-time sample of Google search data, has been publicly available since 2006. Several health-related studies have used Google Trends to measure the interest in infectious diseases and the disease awareness of the general public [18-21]. Google Trends has played a major role in the emerging field of infodemiology, the study of electronically transmitted medical information for the purpose of public health [22]. While there is no absolute method to measure public interest in COVID-19, Google search data has been leveraged in prior research studies as a correlate [14,23,24].

Countries that had prior experience with SARS (and have largely contained COVID-19) instituted robust public health campaigns. These campaigns were targeted to increase public interest in containment and mitigation policies. Increased public interest is thought to be correlated with increased attention and willingness to participate in strategies to reduce person-to-person transmission [9,10,25].

Using Google Trends search queries as a proxy, we examined the US level of interest in COVID-19 during the critical time when containment and mitigation strategies were first being employed. We analyzed how the number of positive SARS-CoV-2 cases affected Google searches in the United States. Lastly, we compared public interest in COVID-19 in the US and Italy to that in countries and regions that have focused on public education as a key strategy, namely Singapore, Hong Kong, and the Republic of Korea, who delivered guidance through traditional print media, broadcast media, social media, and other novel methods [25,26].

## Methods

### Study Tools

This was a retrospective study of the public online search interest in COVID-19 in the early months of 2020 within the United States during the periods of changing case numbers, major news headlines, and implementation of NPIs. Subsections of the United States and other countries and regions were further examined. A variety of tools were used to obtain this understanding.

Google Trends is a publicly available website [27] that allows users to gain an understanding of what the general population is searching for using Google’s search engine. Google searches are stored, anonymized, and processed, and repeat searches are removed [16]. When a user accesses Google Trends, they can extract a value for the search volume of keywords and phrases across specific geographical areas. The output from the tool is converted from the absolute search volume and is reported as a relative volume named “search interest,” which is assigned a numerical value between 0 and 100. We refer to this value as the relative search volume (RSV) [20,28]. An elevated RSV is indicative of a higher proportion of users searching a topic within a set location and time period.

The COVID Tracking Project [29] is a website that aggregates all available COVID-19 testing information in the US for each day. This website collects information from the Department of Health and Human Services in each state. The COVID Tracking Project has been cited by many major news organizations [30,31]. It originally began reporting data on March 4, 2020. Our results were collected by referencing the “US daily 4pm ET” datasheet and using the copy function for the Date and Positive sections. This information was saved as confirmed cases of COVID-19 (Appendix Table 1, Multimedia Appendix 1).

Other tools were used to achieve context in the form of the timeline and major cultural events that surrounded the rise of COVID-19 within the United States. In order to provide societal context, news stories were extracted from the *New York Times* article “A Timeline of the Coronavirus Pandemic” [32] (Appendix Table 2, Multimedia Appendix 1). Google Daily Trends was used to elucidate what drew the attention of the US population in early March. This tool displays the 20 most searched topics daily in the United States, with the ability to review data up to the last 28 days.

### Selection Criteria

The starting date of December 31, 2019, was chosen because a major news headline from Wuhan about an unknown pneumonia appeared on this day [32]. The end date of March 24, 2020, was chosen because this was the most recently accessible date when this project started.

### Study Design

The main topic explored was the search interest in COVID-19. The most commonly searched keyword was chosen to represent this topic. To identify the most searched keyword, on March 30, 2020, we accessed and queried Google Trends for *coronavirus*, *COVID-19*, *COVID*, *SARS*, and *SARS-COV-2*. Filters were used to set a timeline from December 31, 2019, to March 24, 2020, and an additional filter limited the search to the United States. No filter was set for category or search type. The results were graphed over time (Appendix Figure 1, Multimedia Appendix 1). The most searched term, *coronavirus*, was selected for further analysis.

**Figure 1:**
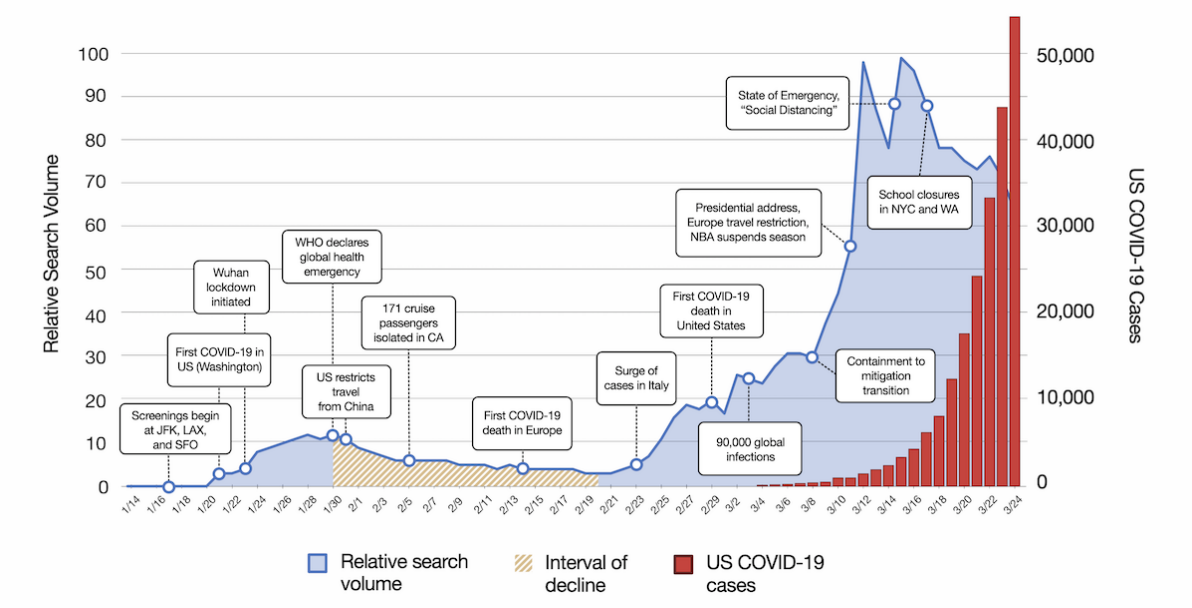
Google Trends RSV (0-100) of the keyword *coronavirus* in the United States graphed over time and during the period of rising COVID-19 cases. Key events are shown on the timeline between January 14, 2020 and March 24, 2020, and the interval decline in search interest is highlighted. COVID-19: coronavirus disease. CA: California. COVID-19: coronavirus disease. JFK: John F. Kennedy International Airport. LAX: Los Angeles International Airport. NBA: National Basketball Association. NYC: New York City. SFO: San Francisco International Airport. US: United States. WA: Washington. WHO: World Health Organization.

### Subgroup Design

A secondary analysis of the relationship between the RSV of *coronavirus* over time in areas with significant outbreaks was performed. Throughout early 2020, New York City, NY, and Seattle-Tacoma, WA, experienced high levels of disease burden within the United States [33-36]. On March 31, 2020, we accessed and queried Google Trends for *coronavirus*. Filters were set for the above timeline, and the location filter was set to New York City, NY. No other filters were set, and the results were downloaded. The same process was repeated with the location filter for Seattle-Tacoma, WA. For another comparison, countries and regions with similar first case dates were chosen for comparison, including Italy, Singapore, Republic of Korea, and Hong Kong. Using the same steps as above with the respective location filters set, the keyword was searched in the local language (Appendix Table 3, Multimedia Appendix 1), and the results were downloaded. In the Daily Search Trends section, we extracted information on the most popular searches from March 3 to March 14.

### Outcome Measures

The primary outcome measure was evaluating the relationship between search interest in COVID-19, major news events, and positive cases of SARS-CoV-2 (Figure 1). Secondary outcome measures included an analysis of the search interest within areas of the United States experiencing high disease burden, an analysis of search interest in specific foreign countries, and an examination of the major search topics in the United States in early March.

### Study Analysis

The RSVs for *coronavirus* were tabled alongside the numbers of COVID-19 cases (Appendix Table 1, Multimedia Appendix 1). The RSV for *coronavirus* was graphed alongside major COVID-19 news headlines and cases of COVID-19 to examine the relationship between the RSV and major events as the number of COVID-19 cases increased (Figure 1). To further analyze the relationship between the number of COVID-19 cases and the RSV of *coronavirus*, the cases of COVID-19 data were linearized using logarithmic transformation with a base of 2. Log2(cases of COVID-19) was then graphed against the RSV for *coronavirus*. A linear relationship was assumed, and a model was fit. Using the Excel data analysis toolkit (Microsoft Corporation), the relationship was analyzed via linear regression. To describe the linear regression, descriptive statistics, including the Pearson coefficient, mean, standard error, and 95% CI, were calculated.

### Subgroup Analysis

**Figure 2:**
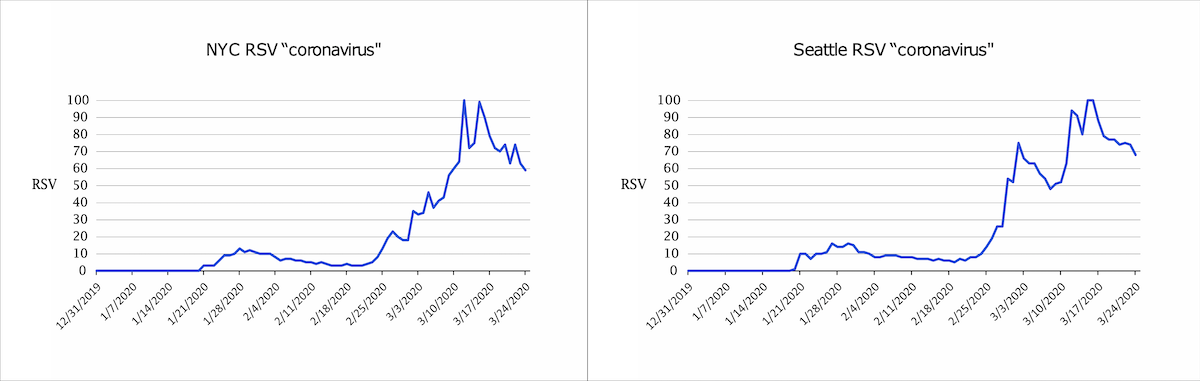
Google Trends RSV (0-100) of the keyword *coronavirus* in New York City, NY and Seattle-Tacoma, WA from December 31, 2019 to March 24, 2020. RSV: relative search volume.

**Figure 3:**
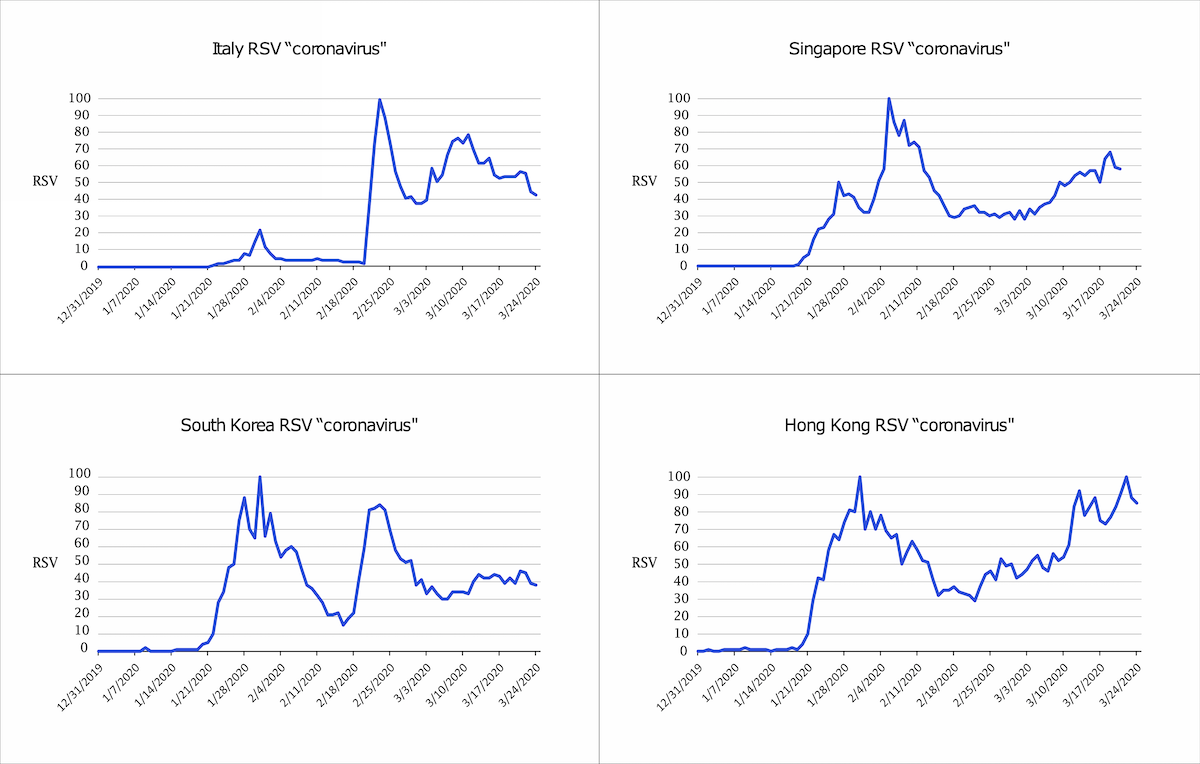
Graphs of Google Trends RSVs (0-100) for the keyword *coronavirus* vs time from December 31, 2019 to March 24, 2020 in Italy, Singapore, South Korea, and Hong Kong. RSV: relative search volume.

**Figure 4:**
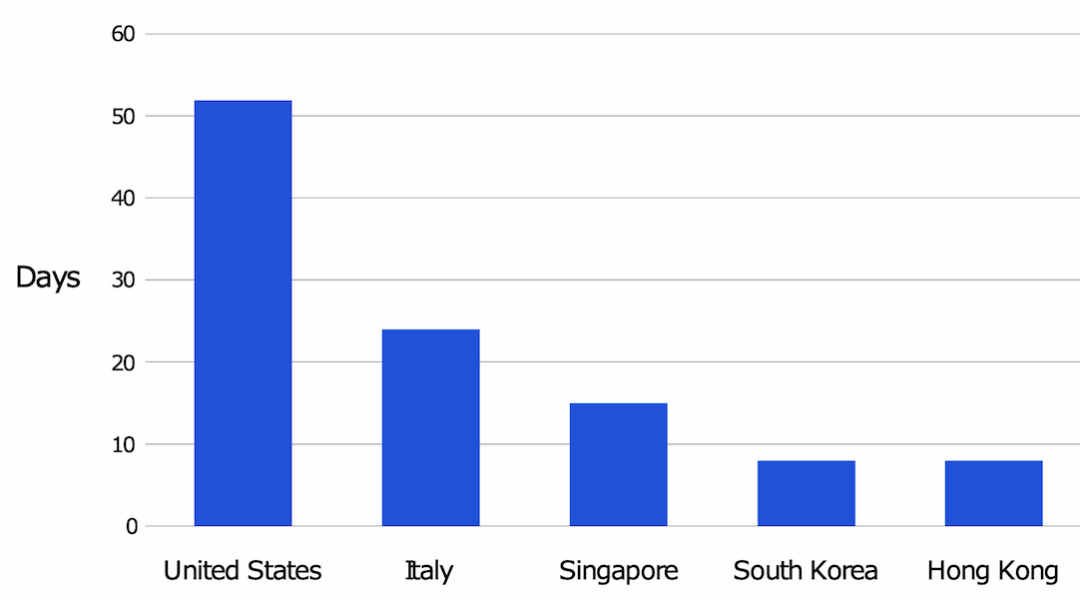
Number of days from the first COVID-19 case until the relative search volume on Google Trends for the search term *coronavirus* reached >90 in the United States, Italy, Singapore, South Korea, and Hong Kong.

The results of the RSV of *coronavirus* over time were graphed for New York City and Seattle-Tacoma (Figure 2) as well as for foreign countries (Figure 3). The first date that the RSV was >90 was recorded alongside the date on which the first case was reported and the time between those two dates (Appendix Table 4, Multimedia Appendix 1). The time between the first case and RSV >90 in each location was then graphed (Figure 4). Top searches for every day leading up to and following March 11 were recorded in a table (Appendix Table 5, Multimedia Appendix 1). Top 5 searches on March 11 were also recorded (Appendix Table 6, Multimedia Appendix 1).

## Results

### Primary Results

**Figure 5:**
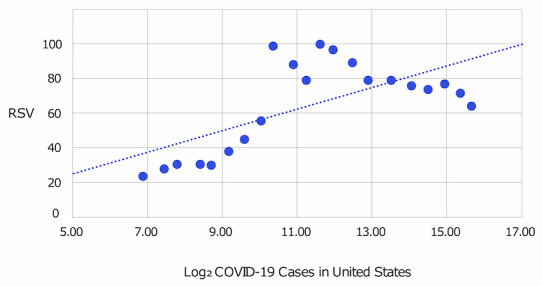
Cases of COVID-19 data linearized using logarithmic transformation with base of 2 graphed against the RSV for the Google search term *coronavirus*. A linear relationship was assumed, and a model was fit. RSV: relative search volume.

The RSV for coronavirus remained below 12 throughout all of January and most of February 2020. It began to rise at the end of February and rose further between March 9 and March 12; Figure 1 demonstrates an increase in the RSV for *coronavirus* within the United States to 99. Figure 5 shows the relationship between Log2(cases of COVID-19) and the RSV for *coronavirus*.

To describe the linear regression, descriptive statistics including Pearson coefficient, t-statistics, standard error, and 95% confidence intervals were calculated (Table 1). There is a significant positive correlation between the two with the equation F(RSV(coronavirus)) = 6.32(Log2(cases of COVID-19)) – 6.97 and R²=0.445. The X variable has a *P* value <.001 and a 95% CI of 2.93-9.71.

**Table 1:** Descriptive statistics of the linear regression of Log2(cases of COVID-19) and the RSV for the search term coronavirus.

Variable	Pearson coefficient (SE)	*t* value	*P* value	95% CI
Intercept	–6.974 (18.846)	–0.370	.715	–46.418 to 32.471
X variable 1	6.321 (1.621)	3.899	<.001	2.928 to 9.714

### Subgroup Results

Figure 2 shows that in both New York City and Seattle, a small increase in interest occurred in January 2020, followed by a decrease in interest throughout February before reaching an RSV of 100 in mid-March. Figure 4 shows the RSV trend for the search term *coronavirus* over time in other countries. Italy showed lower interest throughout January and February 2020 than Singapore, the Republic of Korea, or Hong Kong. Figure 5 shows the time from the first confirmed case until high levels of public interest were reached, reflected by RSV >90. Appendix tables 5 and 6 (Multimedia Appendix 1) show that March 11, 2020 was the day when the most popular daily search topics in the United States reflected COVID-19–related queries.

## Discussion

### Principal Findings

Using Google Trends data, our results indicate that the initial US level of public interest in COVID-19 was limited and even decreased during a time when containment and mitigation strategies were being implemented. On January 17, 2020, the CDC implemented public health airport entry screenings at airports in San Francisco (San Francisco International Airport), New York (John F. Kennedy International Airport), and Los Angeles (Los Angeles International Airport), with announcements of other international airports to follow [37]. The CDC activated its Emergency Operations Center on January 20 [38]. Despite these measures, until January 21, the RSV for *coronavirus* remained at 0, indicating that the US public had low interest in COVID-19.

On January 21, 2020, the CDC reported the first case of COVID-19 in the state of Washington [39]. By the end of January, the World Health Organization had declared COVID-19 a “public health emergency of international concern,” and the United States had implemented aggressive travel restrictions from countries with significant spread [40]. These announcements resulted in the first modest upward movement of public interest. In February, there was actually a relative decline in public interest from February 1 to February 21. This is surprising considering the events that occurred during that time. The *Diamond Princess* cruise ship, with 428 US citizens on board, was found to have hundreds of cases. European deaths were being reported from COVID-19, and multiple countries started to report outbreaks [41,42].

Even more surprisingly, this February decline in public interest was observed in the Seattle and NYC geographical areas, although both areas were experiencing undetected community transmission during this time [33-36]. A similar February downward trend of public interest in COVID-19 was experienced in Italy, a country that experienced a high epidemiologic trajectory of COVID-19 [24]. We suspect this downward trend in public interest in February contributed to the community transmission that occurred in New York City, Seattle, and Italy in February. The reason for this downward trend of COVID-19 public interest should concern policy makers, as this was a critical time for containment measures requiring the public’s attention.

A dramatic increase of public interest in COVID-19 occurred from March 9 to 12, 2020. Several events could explain this. We discovered that the top searched keywords on March 11 related to *Tom Hanks* and *NBA*, each yielding more than ten million searches each. That day, it was reported that actor Tom Hanks had tested positive for SARS-CoV-2 [43]. Additionally, the National Basketball Association (NBA) suspended all games when one of its players tested positive [44]. The third highest search term for the day was related to *coronavirus symptoms*, yielding more than five million searches. That evening, the President of the United States announced travel restrictions from Europe in his first prime time television address of the pandemic [45]. The President’s name was the top search term the following day, with over five million searches on March 12. It is interesting that societal events were associated with sharp increases in COVID-19 public interest. Based on our compiled search term histories, these events and the accompanying media coverage may have contributed to the culmination of public interest in COVID-19.

A strong correlation exists between positive SARS-CoV-2 cases and increasing RSV. We interpret that COVID-19 public interest increased as more cases were discovered. This correlation shows the importance of diagnostic testing. The delays in diagnostic testing that occurred in the United States were a contributor to delaying public interest [46,47]. However, this does not fully explain the substantial lack of public interest. Before the United States developed a high level of public interest (RSV >90), more than 50 days had passed since the first US case of COVID-19, and there had been thousands of positive cases with multiple deaths [30,31].

One of the most interesting and concerning findings was the lack of early public interest in the United States and Italy compared to countries that were able to contain COVID-19 more effectively. From the first cases of COVID-19 in the United States and Italy, 52 and 24 days passed, respectively, before public interest reached high levels. In Singapore, Republic of Korea, and Hong Kong, public interest reached high levels within 15 days of the first positive COVID-19 case.

An important aspect of containment is isolation and quarantine. These both require significant public education and interest for compliance. One of the main goals of modern quarantine is to reduce transmission by increasing the social distance between persons. This requires the general public to understand the actions to take when they are exposed to a disease or develop symptoms, such as effective separation and duration of quarantine [48]. Policy makers should consider partnering with existing popular digital platforms (Facebook, Twitter, Google, and others) not only to monitor interest but to engage the general public when messaging is rapidly changing.

### Limitations

There are several limitations associated with this analysis. First, the exclusive use of Google Trends as a data set does not comprise all internet search traffic. Google constitutes 72% of search engine activity [15]. The remaining internet search activity is conducted on other search engine platforms and is not represented in our analysis. Second, the presumptive association between RSV and public interest has limitations. While RSV offers an innovative method to approximate public interest and has been utilized in prior research, its accuracy in measuring public interest has not been validated [14,23,24]. Third, given the anonymity of the data that Google Trends makes available to the public, it is difficult to determine which segments of the population may be underrepresented in or excluded from the analysis [49]. Fourth, the search criteria used in this analysis are not standardized and may not encompass all search phrases used by the public, including countries that have different platforms and different communication channels. Finally, with 3 months of search information included, conclusions drawn from this data set must be considered in the context of a longer and continually evolving pandemic event, especially within the context of different SARS-CoV-2 timelines.

### Conclusion

Public interest in COVID-19 was limited until March 12, 2020, when a rapid succession of events brought the disease into full public view. Surprisingly, public interest declined into most of February, even in geographic areas that were experiencing undetected community transmission and during a time when containment strategies were in place. While an inability to perform aggressive testing likely contributed to the low level of interest, other countries with improved control of COVID-19 showed accelerated levels of public interest after their first positive cases.

SARS-CoV-2 is now the third novel pathological HCoV to emerge in a relatively short course of time. At this time, there is no proven vaccine or pharmacological treatment available; therefore, adoption of public health initiatives is critical to curtail spread. Based on our analysis, it is clear that policy makers need to develop novel methods of communicating with the public regarding not only SARS-CoV-2 but other emerging infectious diseases. As popular digital tools continue to become ubiquitous, we propose that policy makers should use them not only to understand public interest but to tailor targeted messaging towards the public.

## Appendix

Multimedia Appendix 1 Supplementary information.

## Abbreviations

CDC: Centers for Disease Control and Prevention
COVID-19: coronavirus disease
HCoV: human coronavirus
MERS-CoV: Middle East respiratory syndrome coronavirus
NBA: National Basketball Association
NPI: nonpharmaceutical intervention
RSV: relative search volume
SARS: severe acute respiratory syndrome
SARS-CoV: severe acute respiratory syndrome coronavirus
SARS-CoV-2: severe acute respiratory syndrome coronavirus 2
