# Supplementary material for: Fluctuation of Public Interest in COVID-19 in the United States: Retrospective Analysis of Google Trends Search Data
Source: JMIR Public Health Surveill. 2020 Jul 17;6(3):e19969. doi: 10.2196/19969 (PMC7371405; doi:10.2196/19969)
Supplement: Multimedia Appendix 1 [file publichealth_v6i3e19969_app1.docx]

# Appendix (Supplement)

# Appendix Figure 1: RSV of COVID-19 Keywords

# Appendix Table 1: Raw RSV daily data from Google Trends and COVID positive cases

# Appendix Table 2: News Headlines

# Appendix Table 3: Localized Search Terms for Foreign locations

# Appendix Table 4: First Case and RSV >90 by Location

# Appendix Table 5: Most Popular Google Searches from March 3 to March 14th

# Appendix Table 6: Most Popular Google Searches on March 11


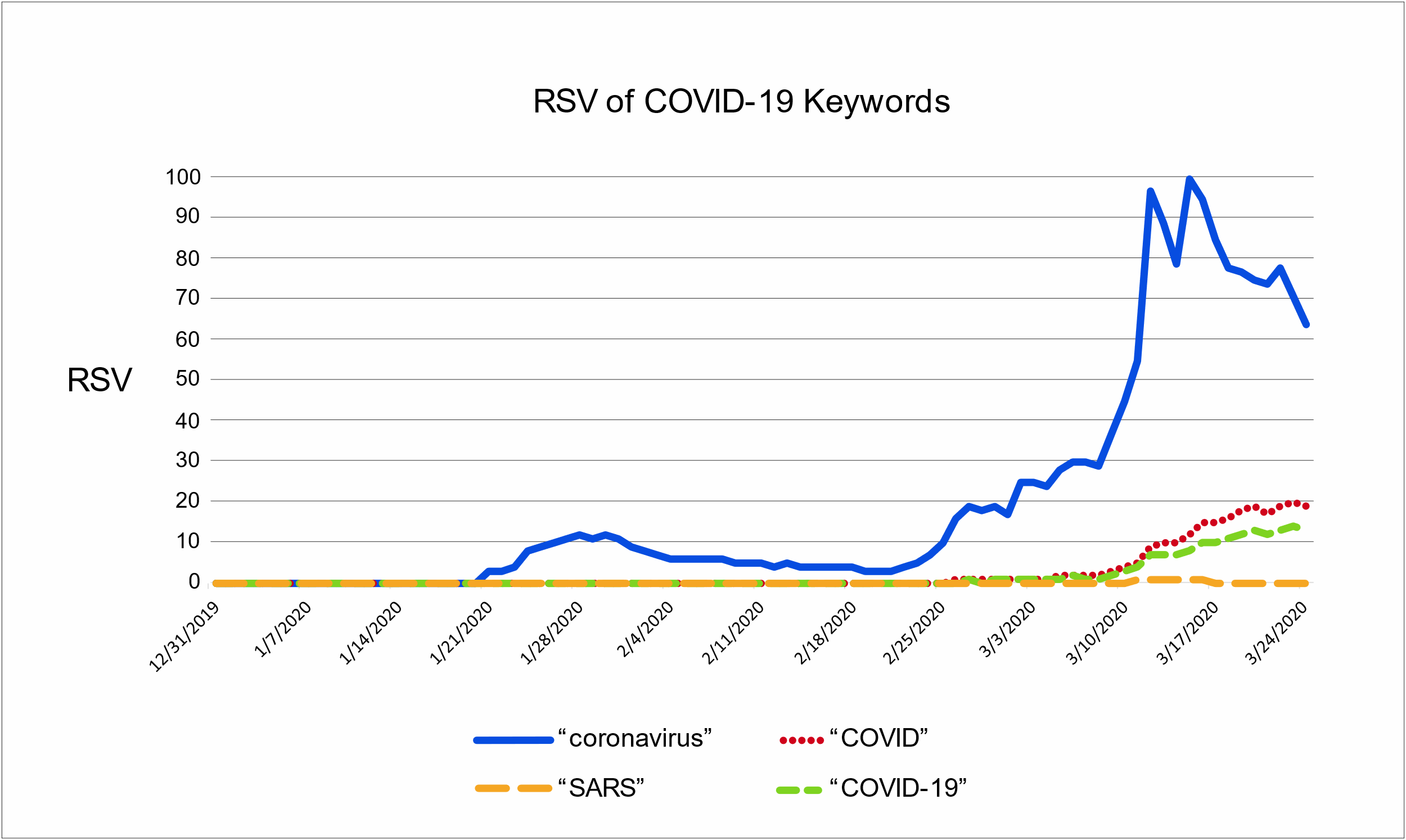


Appendix Figure 1

| Date | "Coronavirus" RSV | COVID Cases | Date | "Coronavirus" RSV | COVID Cases | Date | "Coronavirus" RSV | COVID Cases |
| --- | --- | --- | --- | --- | --- | --- | --- | --- |
| 12/31/19 | 0 |  | **1/29/20** | 11 |  | **2/27/20** | 19 |  |
| 1/1/20 | 0 |  | **1/30/20** | 12 |  | **2/28/20** | 18 |  |
| 1/2/20 | 0 |  | **1/31/20** | 11 |  | **2/29/20** | 20 |  |
| 1/3/20 | 0 |  | **2/1/20** | 9 |  | **3/1/20** | 17 |  |
| 1/4/20 | 0 |  | **2/2/20** | 8 |  | **3/2/20** | 26 |  |
| 1/5/20 | 0 |  | **2/3/20** | 7 |  | **3/3/20** | 25 |  |
| 1/6/20 | 0 |  | **2/4/20** | 6 |  | **3/4/20** | 24 | 118 |
| 1/7/20 | 0 |  | **2/5/20** | 6 |  | **3/5/20** | 28 | 176 |
| 1/8/20 | 0 |  | **2/6/20** | 6 |  | **3/6/20** | 31 | 223 |
| 1/9/20 | 0 |  | **2/7/20** | 6 |  | **3/7/20** | 31 | 341 |
| 1/10/20 | 0 |  | **2/8/20** | 6 |  | **3/8/20** | 30 | 417 |
| 1/11/20 | 0 |  | **2/9/20** | 5 |  | **3/9/20** | 38 | 584 |
| 1/12/20 | 0 |  | **2/10/20** | 5 |  | **3/10/20** | 45 | 778 |
| 1/13/20 | 0 |  | **2/11/20** | 5 |  | **3/11/20** | 56 | 1053 |
| 1/14/20 | 0 |  | **2/12/20** | 4 |  | **3/12/20** | 99 | 1315 |
| 1/15/20 | 0 |  | **2/13/20** | 5 |  | **3/13/20** | 88 | 1922 |
| 1/16/20 | 0 |  | **2/14/20** | 4 |  | **3/14/20** | 79 | 2450 |
| 1/17/20 | 0 |  | **2/15/20** | 4 |  | **3/15/20** | 100 | 3173 |
| 1/18/20 | 0 |  | **2/16/20** | 4 |  | **3/16/20** | 97 | 4019 |
| 1/19/20 | 0 |  | **2/17/20** | 4 |  | **3/17/20** | 89 | 5723 |
| 1/20/20 | 0 |  | **2/18/20** | 4 |  | **3/18/20** | 79 | 7730 |
| 1/21/20 | 3 |  | **2/19/20** | 3 |  | **3/19/20** | 79 | 11719 |
| 1/22/20 | 3 |  | **2/20/20** | 3 |  | **3/20/20** | 76 | 17033 |
| 1/23/20 | 4 |  | **2/21/20** | 3 |  | **3/21/20** | 74 | 23197 |
| 1/24/20 | 8 |  | **2/22/20** | 4 |  | **3/22/20** | 77 | 31879 |
| 1/25/20 | 9 |  | **2/23/20** | 5 |  | **3/23/20** | 72 | 42152 |
| 1/26/20 | 10 |  | **2/24/20** | 7 |  | **3/24/20** | 64 | 51954 |
| 1/27/20 | 11 |  | **2/25/20** | 11 |  |  |  |  |
| 1/28/20 | 12 |  | **2/26/20** | 16 |  |  |  |  |

Appendix Table 1

| News Headlines | |
| --- | --- |
| Date | Event |
| 31-Dec | Wuhan “unknown pneumonia” |
| 11-Jan | First death from the virus |
| 23-Jan | Wuhan, a city of 11 million is placed on total lockdown |
| 30-Jan | WHO declares global health emergency |
| 31-Jan | China travel ban |
| 5-Feb | 171 cruise passengers placed in isolation in California |
| 11-Feb | Named COVID 19 |
| 14-Feb | First death in Europe |
| 21-Feb | Outbreaks in South Korea and Iran |
| 23-Feb | Italy locks down 10 towns |
| 24-Feb | White House petitioned congress for 1.25 billion |
| 29-Feb | First US COVID death in Washington; international travel warning |
| 3-Mar | 90,000 infections are noted in the world |
| 8-Mar | California transition to mitigation |
| 11-Mar | NBA shutdown and Europe travel ban |
| 12-Mar | MLB and NHL shutdown |
| 13-Mar | State of emergency |
| 15-Mar | CDC "social distancing" |
| 17-Mar | Seattle and NYC school closure |
| 22-Mar | NYC stay at home in NYC |

Appendix Table 2

| Localized Search Terms for Foreign locations | | |
| --- | --- | --- |
| Location | Translation for Coronavirus | Exact Search Term Used for this Location |
| Mandarin and Cantonese | 新冠 病毒 | "coronavirus + 新冠 病毒" |
| Korean | 코로나 바이러스 | "coronavirus + 코로나 바이러스" |
| Italian | coronavirus | "coronavirus" |

Appendix Table 3

| First Case and RSV >90 by Location | | | |
| --- | --- | --- | --- |
| Location | First Case Date | RSV >90 Date for "coronavirus" | Public Search Response Time (days) |
| United States | 1/20/2020 | 3/12/2020 | 52 |
| Italy | 1/30/2020 | 2/23/2020 | 24 |
| Singapore | 1/23/2020 | 2/7/2020 | 15 |
| South Korea | 1/20/2020 | 1/28/2020 | 8 |
| Hong Kong | 1/22/2020 | 1/30/2020 | 8 |

Appendix Table 4

| Most Popular Google Searches from March 3 to March 14th | | |
| --- | --- | --- |
| Date | Trending Search | Estimated Number of Searches |
| March 3, 2020 | Super Tuesday results | 10000000 |
| March 4, 2020 | Katy Perry | 2000000 |
| March 5, 2020 | Post Malone | 1000000 |
| March 6, 2020 | Daylight's savings | 2000000 |
| March 7, 2020 | UFC 248 | 2000000 |
| March 8, 2020 | International Women's Day | 1000000 |
| March 9, 2020 | 2020 Election primary results | 2000000 |
| March 10, 2020 | Harvard | 500000 |
| March 11, 2020 | Tom Hanks | 10000000 |
| March 12, 2020 | NBA | 5000000 |
| March 13, 2020 | Coronavirus symptoms | 500000 |
| March 14, 2020 | Pandemic | 10000000 |

Appendix Table 5

| Most Popular Google Searches on March 11 | | |
| --- | --- | --- |
| Date | Trending Search | Estimated Number of Searches |
| 11-Mar | Tom Hanks | 10000000 |
| 11-Mar | NBA | 10000000 |
| 11-Mar | Coronavirus Symptoms | 5000000 |
| 11-Mar | Pandemic | 2000000 |
| 11-Mar | Disneyland | 2000000 |
| 11-Mar | March Madness | 2000000 |

Appendix Table 6
